# NLRP4E regulates actin cap formation through SRC and CDC42 during oocyte meiosis

**DOI:** 10.1186/s11658-024-00580-y

**Published:** 2024-05-10

**Authors:** Li-Ya Shi, Yang Wang, Yan-Jie Yang, Qian Li, Zhi-Xia Yang, Li-Hua Sun, Fu-Qiang Luo, Yu-hao He, Shu-Ping Zhang, Ning Su, Jia-Qi Liu, Ye He, Yi-Chun Guan, Zhao-Lian Wei, Yun-Xia Cao, Dong Zhang

**Affiliations:** 1 Department of Obstetrics and Gynecology, The First Affiliated Hospital of Anhui Medical University, No 218 Jixi Road, Hefei, 230022 Anhui China; 2 Reproductive Medicine Center, Shanghai East Hospital, Tongji University School of Medicine, 551 Pudong South Road, Shanghai, 200120 China; 3 Department of Gynecology, Women’s Hospital of Nanjing Medical University (Nanjing Maternity and Child Health Care Hospital), 123 Tianfei Lane, Nanjing, 210018 China; 4 Center for Reproductive Medicine, The Third Affiliated Hospital of Zhengzhou University, 7 Rehabilitation Front Street, Zhengzhou, 450000 Henan China; 5 NHC Key Laboratory of Study on Abnormal Gametes and Reproductive Tract (Anhui Medical University), No 81 Meishan Road, Hefei, 230032 Anhui China; 6 Key Laboratory of Population Health Across Life Cycle (Anhui Medical University), Ministry of Education of the People’s Republic of China, No 81 Meishan Road, Hefei, 230032 Anhui China; 7 State Key Lab of Reproductive Medicine, Nanjing Medical University, 101 Longmian Ave., Nanjing, 211166 Jiangsu China

**Keywords:** NLRP4E, CDC42, Meiosis, Actin cap, SRC

## Abstract

**Background:**

Members of the nucleotide-binding oligomerization domain, leucine rich repeat and pyrin domain containing (NLRP) family regulate various physiological and pathological processes. However, none have been shown to regulate actin cap formation or spindle translocation during the asymmetric division of oocyte meiosis I. *NLRP4E* has been reported as a candidate protein in female fertility, but its function is unknown.

**Methods:**

Immunofluorescence, reverse transcription polymerase chain reaction (RT-PCR), and western blotting were employed to examine the localization and expression levels of *NLRP4E* and related proteins in mouse oocytes. small interfering RNA (siRNA) and antibody transfection were used to knock down *NLRP4E* and other proteins. Immunoprecipitation (IP)-mass spectrometry was used to identify the potential proteins interacting with *NLRP4E*. Coimmunoprecipitation (Co-IP) was used to verify the protein interactions. Wild type (WT) or mutant *NLRP4E* messenger RNA (mRNA) was injected into oocytes for rescue experiments. In vitro phosphorylation was employed to examine the activation of steroid receptor coactivator (SRC) by NLRP4E.

**Results:**

*NLRP4E* was more predominant within oocytes compared with other NLRP4 members. *NLRP4E* knockdown significantly inhibited actin cap formation and spindle translocation toward the cap region, resulting in the failure of polar body extrusion at the end of meiosis I. Mechanistically, *GRIN1*, and *GANO1* activated *NLRP4E* by phosphorylation at Ser429 and Thr430; p-NLRP4E is translocated and is accumulated in the actin cap region during spindle translocation. Next, we found that p-NLRP4E directly phosphorylated SRC at Tyr418, while p-SRC negatively regulated p-CDC42-S71, an inactive form of CDC42 that promotes actin cap formation and spindle translocation in the GTP-bound form.

**Conclusions:**

NLRP4E activated by *GRIN1* and *GANO1* regulates actin cap formation and spindle translocation toward the cap region through upregulation of p-SRC-Tyr418 and downregulation of p-CDC42-S71 during meiosis I.

**Supplementary Information:**

The online version contains supplementary material available at 10.1186/s11658-024-00580-y.

## Introduction

The nucleotide-binding oligomerization domain, leucine rich repeat and pyrin domain containing (NLRP; or NLR) family, a group of proteins that includes PYD, NACHT, and LRR domains, is large and diverse. NLRP members have been reported to regulate pathophysiological processes, such as inflammation, response against viruses, metabolism, and cell death [1–5]. For example, NLRP3 aggregation induced ASC assembly and activation of downstream inflammation signals, and NLRP3 aggregation in response to diverse stimuli required the enlistment of NLRP3 to dispersed trans-Golgi network (dTGN) [6]. NLRP3 inflammasome was regulated by diverse factors [7–9]. NLRP6 maintains the homeostasis of the gut microbiota to boost immunity to bacterial infection. The mechanism involves NLRP6 binding to viral RNA through Rhx15 (RNA helicase 15), which then triggers the mitochondrial antiviral-signaling protein (MAVS)-dependent type I interferon (IFN) response [10]. Moreover, increasing evidence suggests that NLRs play essential roles in the reproductive system. For example, NLRP5, also known as maternal antigen that embryos require (MATER), is an essential subunit of the subcortical maternal complex (SCMC) and is critical for the zygotes to go through the two-cell stage [11]. MATER is essential for the normal distribution of endoplasmic reticulum (ER) and the homeostasis of Ca^2+^ within oocytes [12]. Mutations of NLRP7 cause reproductive problems, such as abortion, hydatidiform moles, and fetal growth restriction [13]. NLRP14 is a key regulator of the differentiation of primordial germ cell (PGC)-like cells, and NLRP14 knockout causes infertility in mice of both sexes [14]. NLRP14 also regulates calcium homeostasis in early embryonic development by maintaining the stability of mitochondrial Na^+^/Ca^2+^ exchanger (NCLX) through K27-linked ubiquitination [15]. In addition, NLRP14 participates in the immune response and promotes fertilization through negative regulation of cytoplasmic nucleic acid sensing, and mutation leads to hyper-responsiveness to nucleic acids during fertilization and causes male infertility [16]. Human NLRP4 is another NLRP subfamily member that functions in autophagy [17], type I interferon signaling [18], and the IKK/NF-kappa B signaling pathway [19].

Interestingly, mouse NLRP4 has six members named NLRP4A–F. A transcriptome study of the ovaries of Foxo3a-knockout mice characterized NLRP4A, NLRP4B, NLRP4E, and NLRP4F as “female fertility factors,” and their expression patterns in ovaries of 1–14 post-natal days (PNDs) mice were somewhat different. However, their expression levels were upregulated in Foxo3a-knockout ovaries in contrast to control ovaries [20]. Among the four factors, NLRP4F has been characterized as a new subunit of SCMC and as being essential for the formation of cytoplasmic lattices (CPL) and the normal distribution of organelles in mouse oocytes. NLRP4F depletion caused reduced fertility and delayed the development of preimplantation embryos in female mice [21]. The level of NLRP4E was increased the most at PND 14 in the ovaries of Foxo3a-knockout mice compared with the other three factors [20]. However, until now, only one study showed that NLRP4E was important for preimplantation embryo development [22], no other studies address how NLRP4E functions.

On the basis of the expression profile of NLRP4E and the change in expression between control and Foxo3a-knockout ovaries, we hypothesized that NLRP4E may be a typical maternal factor essential for oocyte meiosis. We found that NLRP4E played an essential role in oocyte meiosis by regulating the key actin cap proteins steroid receptor coactivator (SRC) and cell division control protein 42 homolog (CDC42).

## Materials and methods

### Chemicals, reagents, and animals

All chemical reagents were purchased from Millipore Sigma (USA) unless otherwise stated. The mice (females, 3 weeks old) from the Cancer Institute (ICR) were provided by the Beijing Vital River Corporation. All experiments were conducted with the approval of the Labor Protection and Utilization Committee of Nanjing Medical University (approval Number: IACUC-1903028) and Anhui Medical University (Approval No., LLSC-20232253), and the committees follow the rules of the Basel Declaration.

### Antibodies

Information for all commercial primary and secondary antibodies is included in Additional file 1: Table S1.

Rabbit polyclonal anti-NLRP4E antibody was isolated and purified through an immunogen-bound affinity column by Abclonal (Batch No: E1174, Wuhan, China). The immunogen sequence was 75–323 AA in NLRP4E. Rabbit polyclonal anti-phospho-NLRP4E antibody was isolated and purified through immunogen-bound affinity column by ZooNBIO (Nanjing, China), and the immunogen sequence was GIMDSDI(PSer)(PThr)LLD (corresponding to 422–433 AA in NLRP4E).

### Oocyte collection and in vitro culture

The oocytes were collected from the ovaries of 3-week-old ICR female mice. CO_2_-anesthetized mice were sacrificed by cervical disassociation; the ovaries were removed and placed in Hepes medium containing 2.5 μM milrinone and 10% fetal bovine serum. Complexes containing oocytes and granular cells were removed from the antral follicles in ovaries using a hypodermic needle, and the bare oocytes were then separated from the complexes. Next, samples of 50 oocytes were placed in 100 μL minimum essential medium (MEM)+ (minimum essential medium plus 3 mg/mL BSA, 0.2 mM penicillin/streptomycin, 0.01 mM EDTA, and 0.23 mM Na-pyruvate) and covered with mineral oil (Millipore Sigma). The culture settings were 37 °C, 5% O_2_, and 5% CO_2_. Before the experimental treatment, milrinone at 2.5 μM was added to all media to inhibit the onset of meiosis.

### In vitro fertilization (IVF)

Epididymal sperm from B6-DBA2 F1 male mice (10–18 weeks old) were incubated for 1 h in 1 mL MEM+, and then 10 μL of the solution containing 5–10 × 10^6^ sperm/mL, was added to 490 μL MEM+ containing the oocytes. After 5 h, the remaining sperm cells on the surface of the oocytes were removed using a pipette. After 4 h, the oocytes were stained with TUB1A antibody and phalloidin, and the rate of normal fertilization was determined by the presence of the typical two pronuclei.

#### NLRP4E knockdown in oocytes through siRNA

The NLRP4E template sequences for the siRNA are listed in Additional file 1: Table S2. The siRNA was constructed, annealed, and purified using Promega’s T7 in vitro transcription kit (Cat #: P1700, USA). The purified siRNAs were kept at −80 °C. The final small interfering RNA (siRNA) was a mixture of siRNAs corresponding to four different regions at the same final concentration of 5 μM.

For NLRP4E knockdown in oocytes by siRNA, we used Millipore’s siRNA transfection system (Cat #: N2913, USA) following the method described in our previous report. During siRNA processing (usually 36–44 h), 2.5 μM milrinone was added to inhibit meiosis.

### Protein knockdown through antibody transfection

Antibodies used for transfection needed to be free of antiseptic. To this end, the original buffer was diluted over 10^4^ fold using a new buffer [phosphate buffered saline (PBS)/50% glycerol] through repeated centrifugation (5000 rpm) in a filter column with a 100-KDa cutoff.

A Chariot™ protein delivery kit (Active Motif, cat #: 30025, USA) was employed to knock down GRIN1, GNAO1, and SRC. Briefly, two 1.5 mL vials, one containing 1 µL Chariot and 5 µL dd (double-distilled) H_2_O and the other with 1 µg antibody and PBS (6 µL final volume) were prepared. Next, the two vials of solution were gently mixed for 30 min at room temperature (RT) to form the transfection complex, and then the complex was added to a 100 µL MEM+ drop including 50 oocytes. At 12–14 h later, oocytes were cleansed to discard MEM+ that contains the complex. After 2–3 h “rest,” another 1–2 times of transfection were done for the good efficacy of the knockdown.

### Assay of mitochondrial distribution and ATP level

To analyze the distribution of mitochondria, oocytes were incubated with 100 nM mitochondria staining solution (Thermo, Cat #: M7512) for 30 min.

To analyze ATP level, the oocytes were first lysed with RIPA buffer and then tested with an enzyme labeling device (BioTek, USA).

### Rescue experiment

The in vitro transcriptional template of mRNA was obtained by cloning the entire enhanced green fluorescent protein (EGFP)-*Src* fragment into pBluescript II(+) and linearizing with FsiI. EGFP-*Src* mRNA was obtained by using a T3 in vitro mRNA transcription kit (Ambion, Cat #: AM1348, USA) and purified with a mini RNA purification kit (Qiagen, Cat #: 74004, Germany). After NLRP4E knockdown, the oocytes were injected with approximately 7 pL (500–1000 ng/mL) of EGFP-*Src* mRNA, then maintained for 16 h in the GV phase and analyzed in subsequent experiments.

### Coimmunoprecipitation (Co-IP)

To verify the interaction between NLRP4E and SRC, Rabbit anti-NLRP4E or anti-SRC antibody was separately bound onto 15 μL of protein A/G (Yeason, cat #: 36417ES, China) and resuspended in 250 μL of IP buffer on a rotator at 4 °C. Meanwhile, the oocytes were subjected to ultrasonication in IP buffer, and then precleaned using protein A/G 4 h at 4 °C. The precleaned oocyte lysate supernatant was incubated with protein A/G-bound NLRP4E or SRC antibody ON at 4 °C. Finally, the protein A/G immune complexes were washed three times with IP buffer (10 min per wash), and western blot samples were prepared. NLRP4E reaction samples and SRC samples were loaded in parallel onto a sodium dodecyl-sulfate polyacrylamide gel electrophoresis (SDS-PAGE) gel and then detected by NLRP4E or SRC antibodies. During the entire process, protease inhibitors and phosphatase inhibitors (Yeason) were included according to the instructions.

The same procedure was applied to the interaction between the other two proteins considered in this study.

### Identification of NLRP4E interacting proteins

Control immunoglobulin G (IgG) or NLRP4E IP was treated as above to bait potential interacting proteins, and then Protein A/G immunocomplexes were constructed into protein samples. Then, Control IP and NLRP4E IP samples were loaded in parallel onto an SDS-PAGE gel and silver stained.

For the silver staining, the gel described above was first fixed in a 40% ethanol, 10% acetic acid solution ON at 4 °C, then placed in sensitizing solution (fresh-made; 6.8% sodium acetate, 30% ethanol, 0.314% Na_2_S_2_O_3_·5H_2_O, and 0.2% Na_2_S_2_O_3_) for 30 min at RT, and washed three times in water (5 min per wash). Next, the gel was placed in staining solution (fresh-prepared, 0.02% of 37% formaldehyde solution, 0.25% AgNO_3_) for 20 min at RT, washed with water for 2.5 min, and placed in developing solution (0.02% of 37% formaldehyde solution, 2.5% NaCO_3_) for about 5 min. Finally, the developing reaction was terminated with stopping solution (0.4% glycine) for 10 min.

To identify proteins interacting with NLRP4E, we selected the bands with higher intensity in the NLRP4E lane than in the control lane, cut both bands in parallel (proteins identified in control bands were used to subtract the proteins in NLRP4E bands), and then sent these to Bio-Tech Pack, Beijing, China, for mass spectrometry.

### In vitro phosphorylation assays

SRC-EGFP-Strep II or NLRP4E-TagRFP-Flag purified as above were mixed in an equal molar ratio (Fig. 7D) or a gradually increased NLRP4E molar ratio (Fig. 7E) and incubated for 20 min at RT. The reaction product was examined by western blotting. The reaction buffer was BRB80 (with 1 mM ATP, 5 mM DTT, and 10% glycerol).

### Sample grouping, data collection, and analysis

Any oocytes for experimental treatment needed to be of high quality (fully grown oocytes, regular diameter, the zona pellucida tightly connected to the oocyte membrane). Any low-quality or unhealthy oocytes were not employed in the analysis.

During the experiments, including grouping, data collection, and data analysis, we used the blind method whenever possible. Data collection, data analysis, and data documentation (into Word or Excel files) were carried out by separate participants.

Before the experiment, each oocyte was assigned randomly to an independent replicate or group. Each data point was obtained and analyzed using blind choice.

### Data analysis and statistics

Each experiment had at least three repeats. Image J (NIH, USA) was employed to analyze confocal images. Individual data points in the graphs are shown as mean ± standard error of the mean (SEM). For statistical analysis of differences between groups, we used Student’s *t*-test (Excel, Microsoft, USA). For statistical analysis of three or more groups, we used one-way nonparametric analysis of variance (ANOVA; GraphPad, USA). Values with *p* < 0.05 were considered significant.

## Results

### NLRP4E is an oocyte-predominant protein

**Fig. 1:**
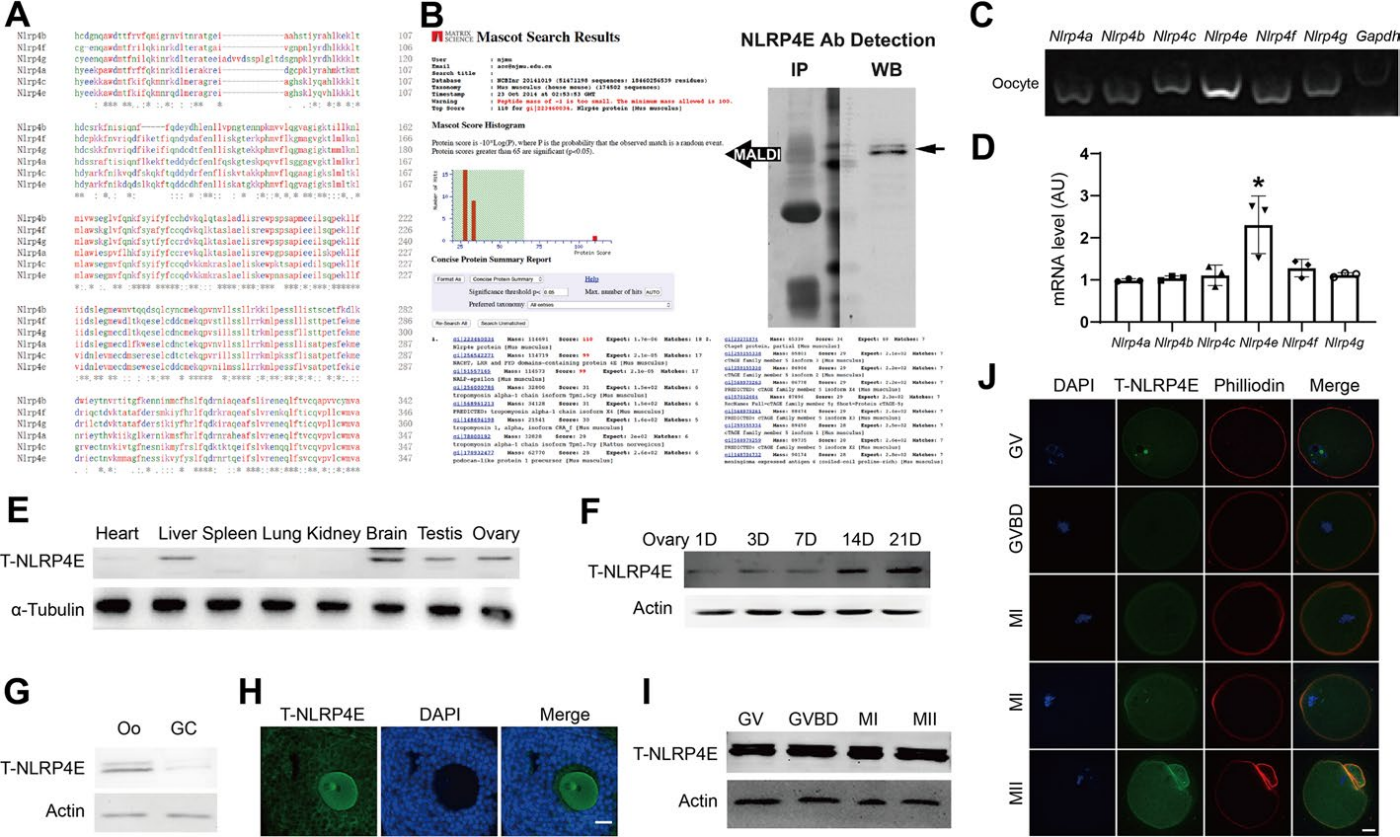
NLRP4E is an oocyte-predominant protein. **A** The AA sequence alignment between NLRP4 family members, including the 75–323 AA region in NLRP4E for construction of the polyclonal antibody used in this study. **B** Verification of the specificity of NLRP4E polyclonal antibody. We used NLRP4E antibody to perform immunoprecipitation followed by SDS-PAGE and silver staining. The gel band (left) corresponding to the NLRP4E blot band (right) was cut and sent for MALDI. The results show that the identified protein with maximum intensity was NLRP4E. **C**, **D** RT-PCR shows that NLRP4E mRNA was the richest among NLRP4 family mRNAs. **E** Western blot showing that NLRP4E was predominantly expressed in the brain, liver, testis, and ovary. **F** Western blot showing that the NLRP4E level gradually increased in mouse ovaries from post-natal day (PND) 1 to 21 and reached a peak on PND 21. **G**, **H** Western blots (**G**) and immunofluorescence (**H**) show that NLRP4E was more predominant in oocytes than in GCs (granular cells). **I** Western blots show that the NLRP4E level remained constant during oocyte meiosis. **J** Immunofluorescence shows that NLRP4E accumulates at the cell membrane during oocyte meiosis. DAPI is shown in blue, NLRP4E in green, and actin filaments in red.  GV, germinal vesicle; GVBD, germinal vesicle breakdown; MI, metaphase I; MII, metaphase II. Scale bar, 50 μm in H, 20 μm in J. * indicates p  < 0.05

As stated above, we hypothesized that NLRP4E could be a typical maternal factor essential for female meiosis. We used a custom-made NLRP4E antibody (Abclonal, Wuhan) comprising 75–323 AA (we tried several commercial antibodies, but they did not work well). This region shared quite a high similarity with other mouse NLRP4 members (Fig. 1A). To verify the specificity of the NLRP4E antibody, we performed IP with this antibody and cut the gel region corresponding to the NLRP4E blot band and sent these for matrix-assisted laser desorption/ionization (MALDI). The results showed that the top three identified proteins were all NLRP4E (Fig. 1B). Next, we found that the abundance of NLRP4E mRNA was the highest among NLRP4 family members (Fig. 1C and D), and NLRP4E was relatively rich in the ovary, testis, liver, and brain (Fig. 1E).

The western blots showed that the NLRP4E level increased gradually from PND 1–21, being particularly high at PND 21 (Fig. 1F), and NLRP4E was more abundant in oocytes compared with GCs (Fig. 1G and H). The NLRP4E level remained stable throughout oocyte meiosis (Fig. 1I), and NLRP4E accumulated at the membranes of oocytes during meiosis (Fig. 1J). These results suggest that NLRP4E may be an essential maternal factor for oocyte meiosis.

### NLRP4E knockdown affects oocyte meiosis and quality

**Fig. 2:**
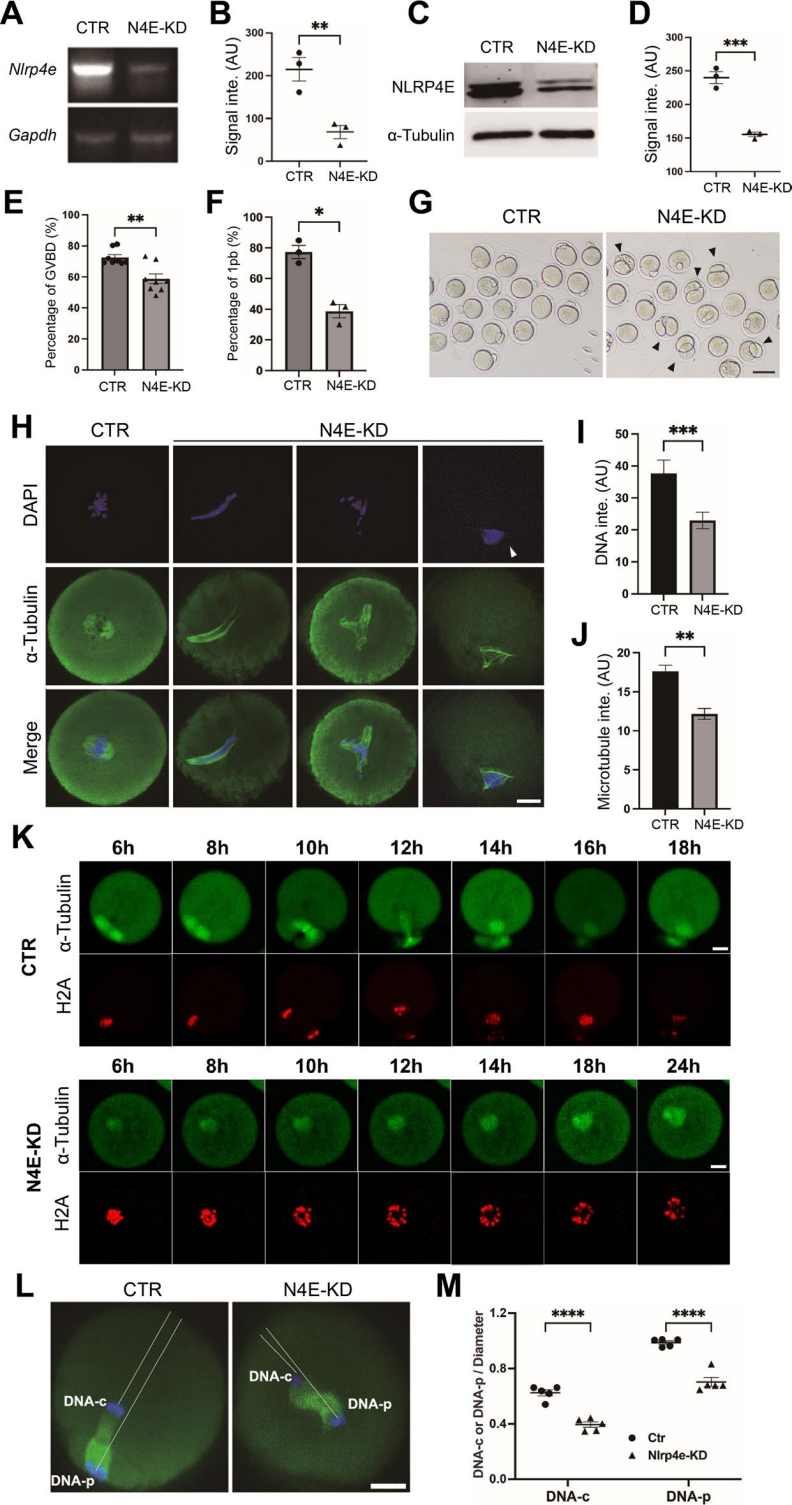
NLRP4E knockdown disrupts oocyte meiosis. **A**, **B** RT-PCR and quantification showing that the NLRP4E mRNA level was significantly reduced by siRNA knockdown. **C**, **D** Western blotting shows that the NLRP4E level was significantly reduced by siRNA knockdown. **E**–**G** Quantification shows that NLRP4E knockdown significantly reduced the percentages of GVBD (**E**) and 1pb (the first polar body, **F** and **G**). **H**–**J** Immunofluorescence analysis shows that NLRP4E knockdown caused significant disruption of chromosomes and spindle microtubules. DNA is shown in blue and microtubules are in green. **K** Slices from live imaging show that in some oocytes with severe NLRP4E knockdown, the chromosomes failed to segregate at the end of meiosis. EGFP-tubulin mRNA and TagRFP-histone were injected into oocytes to label microtubules and chromosomes. **L**, **M** Immunofluorescence analysis shows that in oocytes with medium NLRP4E knockdown, the chromosomes could segregate at the end of meiosis, but spindle translocation toward the cortex was significantly blocked. Scale bar, 80 μm in **G** and 20 μm in other panels.  * indicates p  < 0.05; ** indicates p  < 0.01; *** indicates p  < 0.001; **** indicates p  < 0.0001

We first performed NLRP4E knockdown (KD) by siRNA transfection and analyzed the meiotic phenotype in IVM oocytes. Both RT-PCR and western blots showed that NLRP4E had been eliminated efficiently by siRNA (Fig. 2A–D). We found that NLRP4E KD greatly reduced the ratio of GVBD and pb1 extrusion (Fig. 2E–G). The chromosomes were more dispersed, and the spindles were severely disorganized (Fig. 2H–J). Live imaging of RFP-histone and GFP-tubulin-injected oocytes showed that NLRP4E KD impeded homologous chromosome segregation and polar body extrusion (Fig. 2K, Additional file 2: Movie S1). Even in those NLRP4E-KD oocytes with successful homologous chromosome segregation, chromosome translocation toward the cortex was blocked (Fig. 2L and M).

**Fig. 3:**
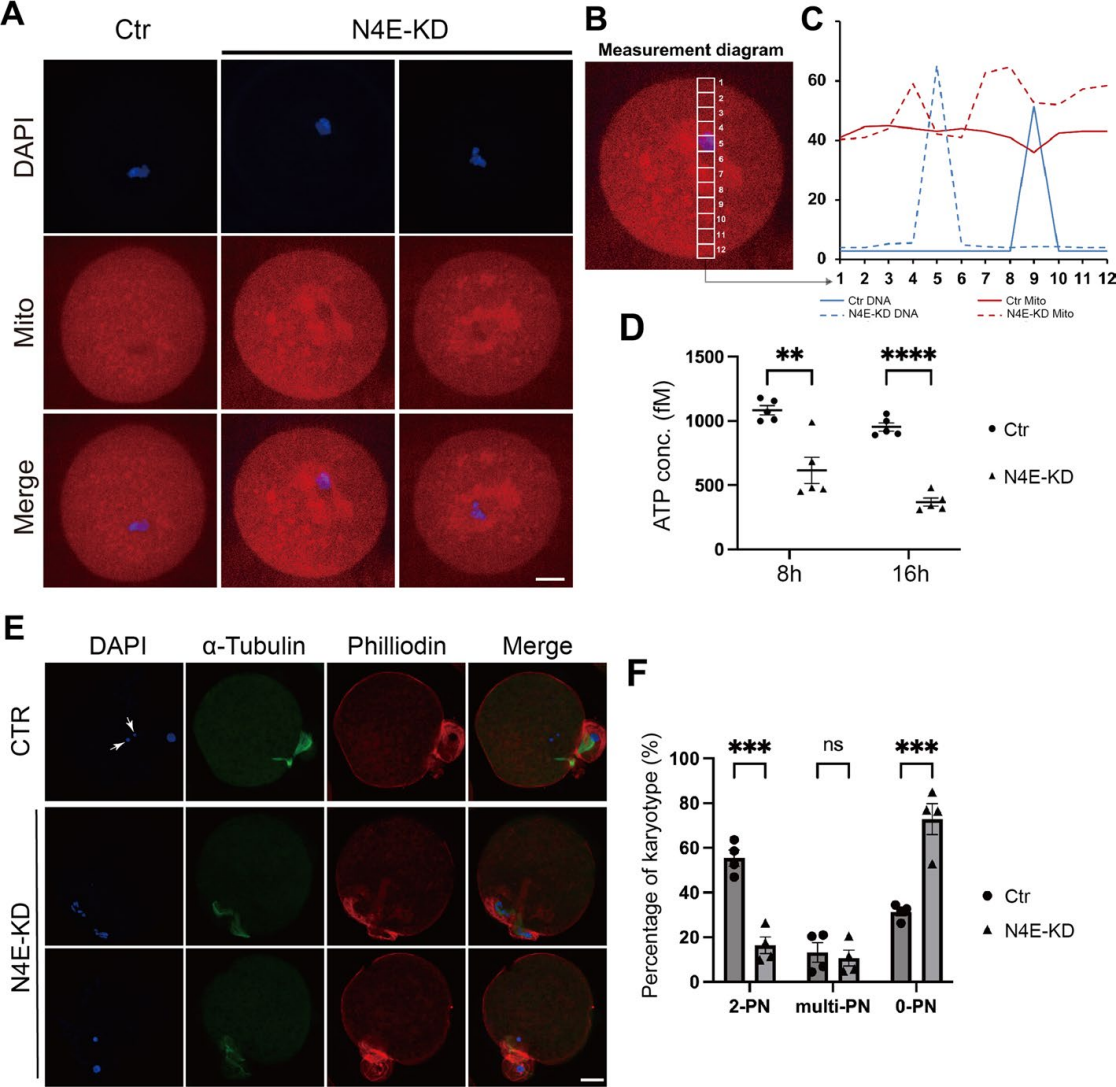
NLRP4E knockdown impairs oocyte quality. **A**–**D** NLRP4E knockdown caused severe mitochondrial aggregation (**A**–**C**), and the ATP concentration was significantly decreased in NLRP4E-knockdown oocytes (**D**). **E**, **F** NLRP4E knockdown significantly decreased the percentage of normal fertilization (2-PN, two pronuclei). Scale bar, 20 μm.  ** indicates p  < 0.01; *** indicates p  < 0.001; **** indicates p  < 0.0001

We next examined how the abnormal meiosis affected the health status of NLRP4E-KD oocytes. Mito tracker staining showed that NLRP4E KD caused abnormal mitochondrial aggregation (Fig. 3A–C), and the ATP concentration within the NLRP4E-KD oocytes was also significantly reduced (Fig. 3D). These results indicated that the oocytes were severely damaged; consequently, IVF showed that NLRP4E KD had a significantly decreased rate of normal fertility (Fig. 3E and F).

### NLRP4E knockdown disrupts actin cap formation

**Fig. 4:**
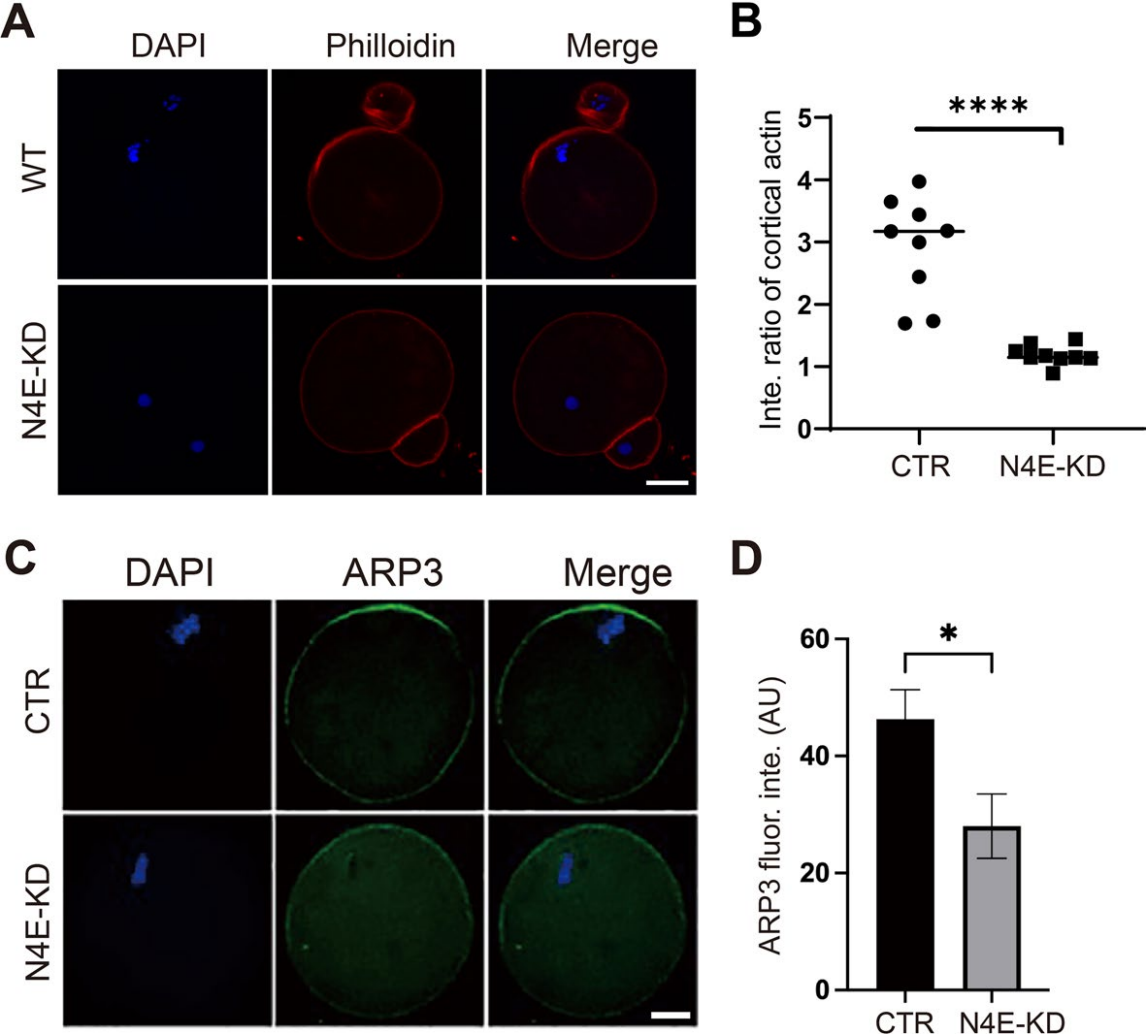
NLRP4E knockdown disrupted actin cap formation. **A**, **B** Phalloidin staining and quantification show that NLRP4E knockdown caused a significant decrease in the intensity of actin filaments at the cap region. DNA is shown in blue, and phalloidin in red. **C**, **D** Immunofluorescence analysis shows that NLRP4E knockdown caused a significant decrease in the intensity of ARP3 at the cap region. DNA is shown in blue and ARP3 in green. Scale bar, 20 μm.  * indicates p  < 0.05; **** indicates p  < 0.0001

The abnormal chromosome translocation suggested that NLRP4E’s function could be correlated with actin cap formation or dynamics. The results showed that NLRP4E KD decreased the actin intensity in the actin cap region during polar body extrusion (Fig. 4A and B).

Arp2/3 complexes have been reported to affect spindle migration, asymmetric division, and cytokinesis during oocyte meiosis by regulating actin cap formation [23–25]. Accordingly, NLRP4E KD decreased the Arp3 intensity in the actin cap region during chromosome translocation (Fig. 4C and D).

### NLRP4E is activated through phosphorylation at S429 and T430

**Fig. 5:**
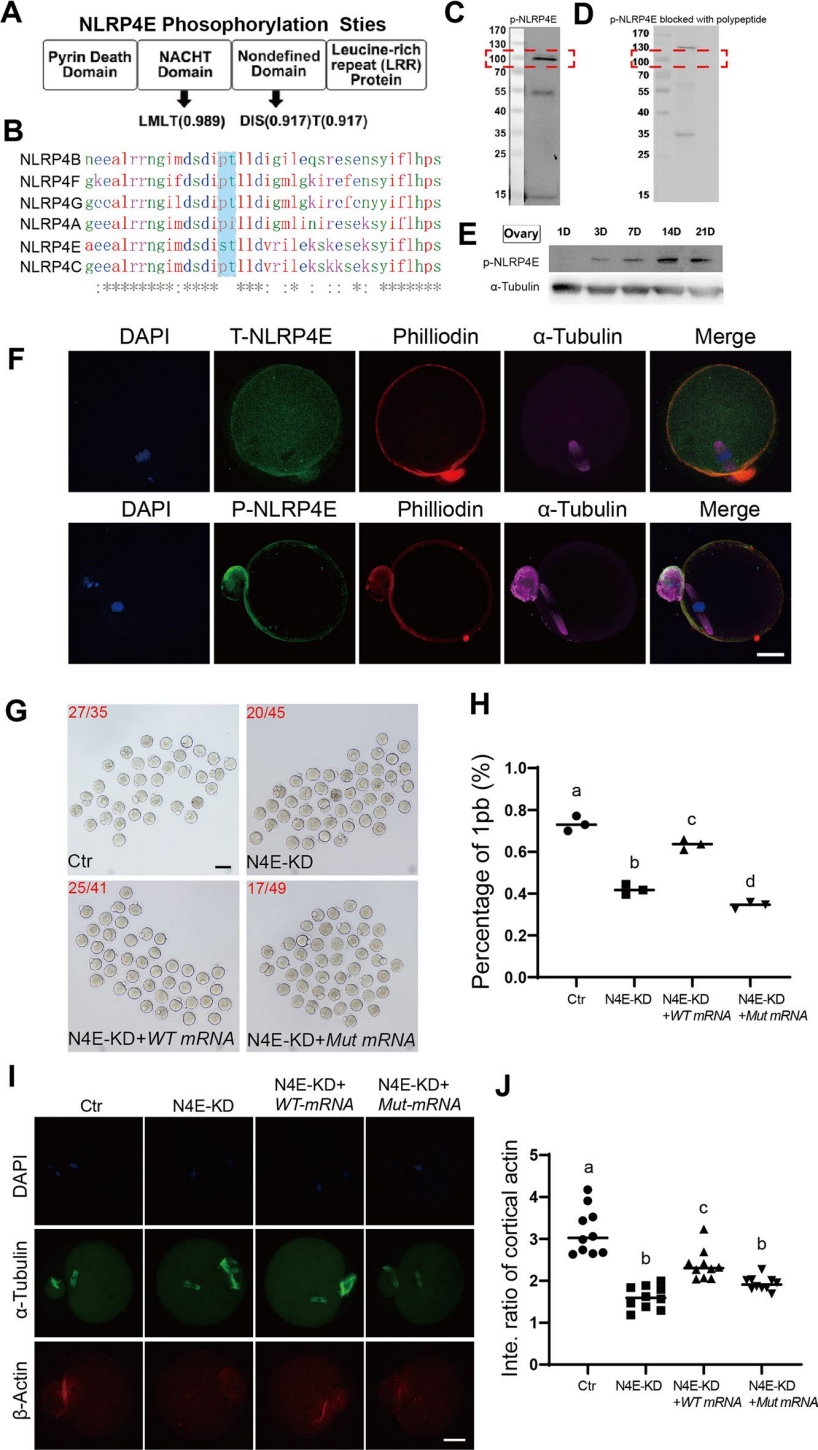
NLRP4E is activated through phosphorylation at S429 and T430. **A** and **B** We used NLRP4E antibody to perform IP, followed by phosphor protein enrichment and mass spectrometry. Three phosphor sites were identified, one at Thr165 in the NACHT domain, and the other two at Ser429 and Thr430 in a nondefined domain. Sequence alignment showed that Ser429 and Thr430 were unique among all NLRP4 members; therefore, we chose GIMDSDI(PSer)(PThr)LLD, which included Ser429 and Thr430, for custom-made phospho-specific NLRP4E antibody (p-NLRP4E antibody). **C** and **D** We preblocked p-NLRP4E antibody with GIMDSDI(PSer)(PThr)LLD, then performed western blotting with p-NLRP4E antibody. The correct-size band (red dotted line rectangle) completely disappeared, indicating that the p-NLRP4E antibody was fairly specific. **E** Western blot showed that the p-NLRP4E level gradually increased in mouse ovaries from PND (post-natal day) 1 to 21 and reached a peak on PND 21. **F** Immunofluorescence indicates that compared with NLRP4E that had an even distribution on the oocyte membrane, p-NLRP4E accumulated within the cap region. DNA is shown in blue; T-NLRP4E/p-NLRP4E is in green; actin filaments (phalloidin) are shown in red, and tubulin is in magenta. **G**, **H** In vitro maturation and quantification show that NLRP4E knockdown (KD) significantly reduced the maturation rate (percentage of 1pb). Injection of NLRP4E-WT mRNA significantly elevated the maturation rate, whereas the injected nonphospho NLRP4E-S429A & T430A mutant failed to rescue the reduced maturation rate, and the rate in the mutant group was lower than in the NLRP4E-KD group. **I**, **J** Immunofluorescence analysis shows that NLRP4E knockdown (KD) significantly reduced the intensity of actin filaments at the cap region; injected NLRP4E-WT mRNA significantly elevated the intensity, whereas the injected nonphospho NLRP4E-S429A and T430A mutant failed to rescue the reduced intensity. Scale bar, 80 μm in **G**, 20 μm in other panels. Different lower-case letters in **H**, **J** indicate significant differences between the two groups

From the above results, we concluded that NLRP4E regulates cap formation and dynamics. However, NLRP4E was evenly enriched on the oocyte membrane, leading us to hypothesize that NLRP4E must be posttranslationally modified (for example, by phosphorylation) to relocate to the actin cap region. To verify this, we used NLRP4E antibody for IP followed by phosphoprotein enrichment. We identified several phosphorylation sites and chose S429 and T430 (Fig. 5A), sites that are unique to NLRP4E compared with other NLRP4 members (Fig. 5B), as key sites. Next, we generated a phosphorylation antibody using “GIMDSDI(PSer)(PThr)LLD” and verified its specificity by corresponding non-phosphopeptide blocking and western blotting (Fig. 5C and D).

We subsequently found that the level of phosphorylated NLRP4E (p-NLRP4E) in ovaries increased gradually from PND 1 to PND 21 (Fig. 5E), similar to the expression pattern of total NLRP4E (t-NLRP4E). Next, we found that compared with t-NLRP4E, p-NLRP4E strongly aggregated at the actin cap region (Fig. 5F), indicating its role in regulating actin cap formation and dynamics.

To further verify the importance of S429 and T430 phosphorylation in the function of NLRP4E, we injected in vitro transcribed NLRP4E-WT or S429 and T430 mutant mRNA into NLRP4E-KD oocytes and examined their maturation rate (1pb rate). The results showed that NLRP4E-WT mRNA injection significantly recovered the 1pb rate, while NLRP4E-AA mRNA injection further reduced the 1pb rate compared with that in the NLRP4E-KD oocytes (Fig. 5G and H). Accordingly, NLRP4E-WT mRNA injection significantly increased the actin intensity in the cap region, while NLRP4E-AA mRNA injection did not (Fig. 5I and J).

### GRIN1 and GNAO1 activate NLRP4E, which subsequently regulates SRC and CDC42 phosphorylation and actin cap assembly

**Fig. 6:**
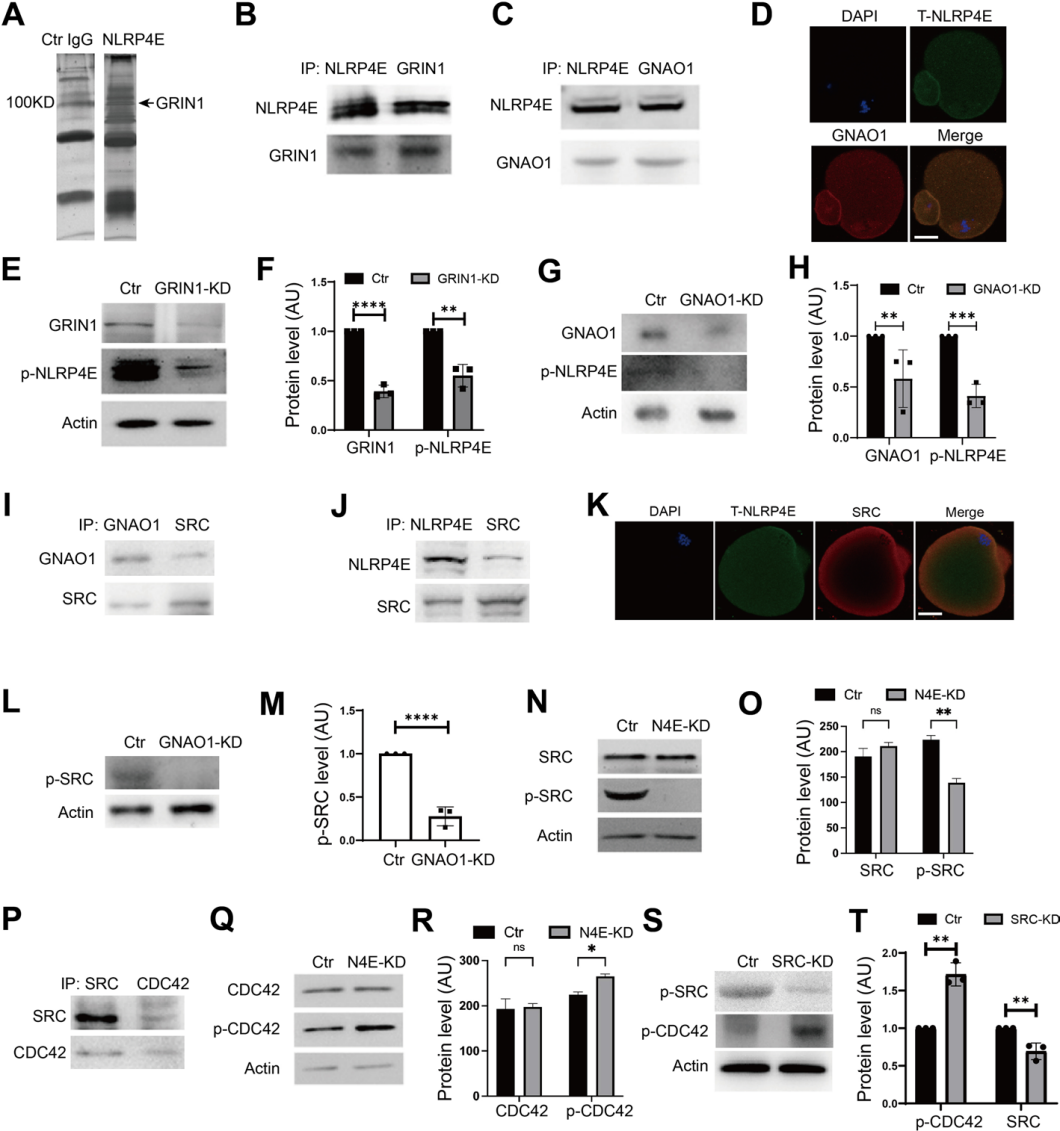
GRIN1 and GNAO1 activate NLRP4E, which subsequently regulates SRC and CDC42 phosphorylation and actin cap assembly. **A** We used NLRP4E antibody to perform IP and MALDI and identified four interacting proteins, one of which was GRIN1. **B** Co-IP and western blotting verified that NLRP4E interacted with GRIN1. **C** Co-IP- and western blotting show that GANO1interacted with NLRP4E. **D** Immunofluorescence shows that GANO1 colocalized with NLRP4E at the oocyte membrane. NLRP4E is shown in green, GANO1 is in red, and DNA is in blue. **E**–**H** Western blots showing that both GRIN1 (**E** and **F**) and GANO1 (**G** and **H**) knockdown significantly reduced p-NLRP4E. **I**, **J** IP-blot showing that both GANO1 (**I**) and NLRP4E (**J**) interacted with SRC. **K** Immunofluorescence showing that NLRP4E colocalized with SRC at the oocyte membrane. NLRP4E is shown in green, SRC is in red, and DNA is in blue. **L**–**O** Western blot showing that both GANO1 (**L** and **M**) and NLRP4E (**N** and **O**) knockdown significantly reduced p-SRC. **P** Co-IP blot showing that SRC interacted with CDC42. **Q**–**T** Western blots showing that both NLRP4E (**Q** and **R**) and SRC (**S** and **T**) knockdown significantly increased p-CDC42. Scale bar, 20 μm.  * indicates p  < 0.05; ** indicates p  < 0.01; *** indicates p  < 0.001; **** indicates p  < 0.0001

To further investigate how p-NLRP4E regulated actin cap formation, we performed IP with the NLRP4E antibody in an oocyte lysate followed by MALDI-TOF-MS and characterized four potential NLRP4E-interacting proteins including G protein-regulated inducer of neurite outgrowth 1 (GRIN1), ribosomal protein S6 kinase alpha-1 (RSK), glycogen phosphorylase, and radial spoke head protein 6 homolog A (RSHL1). Among these four, GRIN1 (Fig. 6A) has been shown to interact with guanine nucleotide binding protein, alpha O (GANO1) and promote the GTP-bound CDC42, the active form of CDC42, at the growth cone of neuronal cells [26], whereas GTP-bound CDC42 is critical for actin cap formation, spindle attachment to the cap, and polar body extrusion during oocyte meiosis I [27–29]. In addition, SRC has been reported to promote actin polymerization [30–32]. Therefore, we thought that NLRP4E could interact with or be regulated by GRIN1 and GANO1.

We first showed that both GRIN1 and GANO1 interacted with NLRP4E (Fig. 6B and C), and NLRP4E colocalized with GNAO1 at the cell membrane (Fig. 6D), while both GRIN1 and GANO1 knockdown significantly reduced p-NLRP4E (Fig. 6E–H). Next, we demonstrated that both GANO1 and NLRP4E interacted with SRC (Fig. 6I and J), and NLRP4E colocalized with SRC at the cell membrane (Fig. 6K), while both GANO1 and NLRP4E knockdown significantly reduced p-SRC (Fig. 6L–O). Next, we found that SRC interacted with CDC42 (Fig. 6P), whereas both NLRP4E and SRC knockdown significantly increased p-CDC42 (Fig. 6Q–T).

### NLRP4E directly binds and phosphorylates SRC

**Fig. 7:**
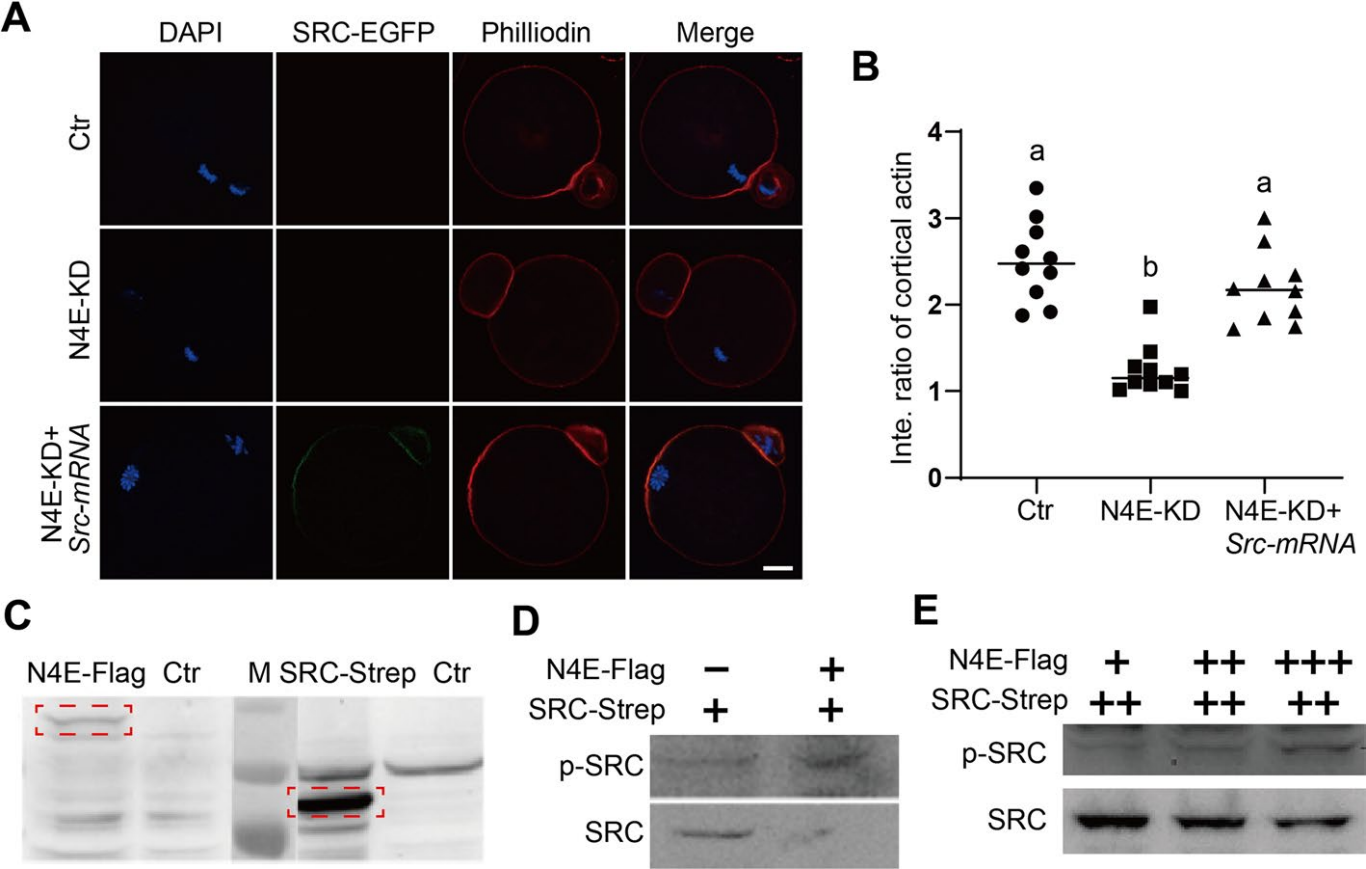
NLRP4E directly binds and phosphorylates SRC. **A**, **B** Immunofluorescence analysis showing that NLRP4E knockdown (KD) significantly reduced the intensity of actin filaments at the cap region; injected SRC-WT-EGFP mRNA significantly elevated the intensity. DNA is shown in blue, SRC-WT-EGFP is in green, and actin filaments (phalloidin) are in red. **C**–**E** In vitro phosphorylation assay and western blot showing that purified NLRP4E-flag could dose-dependently phosphorylate SRC. Scale bar, 20 μm. Different lower-case letters indicate significant differences between the two groups

We further investigated the relationship between NLRP4E and SRC. We found that injection of SRC-mRNA could rescue the cap formation in NLRP4E-knockdown MII oocytes, suggesting that SRC functionally overlapped with NLRP4E in actin cap formation (Fig. 7A and B). Next, we found that in vitro purified NLRP4E could phosphorylate SRC in a dose-dependent pattern (Fig. 7C–E).

## Discussion

In the present study, we characterized the expression, localization, function, activation, and regulatory mechanism of NLRP4E. On the basis of our data, NLRP4E appears to be functionally distinct and is perhaps more important in oocyte meiosis compared with other NLRP (and even other NLRP4) family members.

NLRP4E appeared to be especially important for cap formation and spindle translocation during oocyte meiosis I. Other NLRP and NLRP4 members are important for inflammation, immune responses, metabolism, cell death, the first embryonic cell division, PGC-like cell differentiation, autophagy, CPL formation, and organelle distribution during oocyte meiosis [1–22]. However, none of the NLRP members have been shown to regulate cap formation, spindle translocation toward the cap region, or polar body extrusion during oocyte meiosis. Here, we first showed that NLRP4E knockdown significantly inhibited spindle translocation toward the actin cap; we found that NLRP4E knockdown significantly diminished the intensity of actin filaments and the key cap protein ARP3 within the cap region, and we showed that NLRP4E interacted with and activated the key kinases SRC and CDC42. All these lines of evidence suggested the specific function of NLRP4E.

In the present study, NLRP4E appeared to function through a unique mechanism during oocyte meiosis. We demonstrated that NLRP4E interacted with and directly activated SRC and NLRP4E knockdown significantly reduced p-SRC. SRC has been reported to be important for actin polymerization [30–32], in agreement with our findings. We also demonstrated that NLRP4E interacted with CDC42, and NLRP4E knockdown significantly increased p-CDC42 (Ser71). The activity of CDC42 (GTP-bound form) is negatively regulated by Ser71 phosphorylation, and the constitutively phosphor-mimic form of CDC42, S71E, did not activate CDC42 downstream key actin cap kinase PAK1/2 [33], which is also in agreement with our results.

In addition, we showed that SRC depletion significantly increased p-CDC42 (Ser71), suggesting that SRC positively regulates the activity of CDC42. Various studies have shown that SRC phosphorylation increased the activity of CDC42 [34–36], which is also in accordance with our results.

## Conclusions

**Fig. 8:**
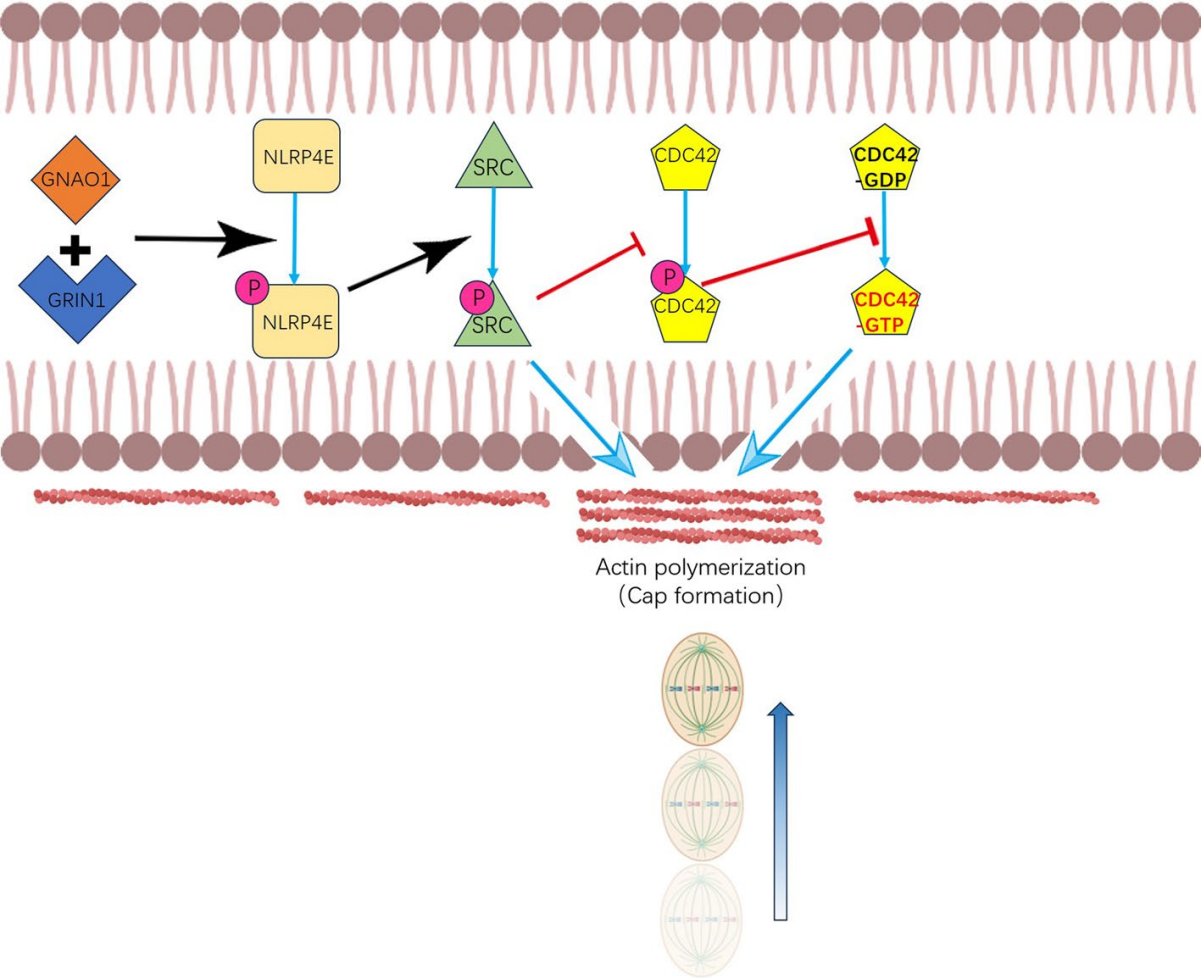
Working Model. Our experimental findings suggested that GRIN1 and GNAO1 phosphorylate NLRP4E at Ser429 and Thr430, and then p-NLRP4E is translocated and accumulates in the actin cap region to activate SRC by phosphorylation at Tyr418. p-SRC could itself promote cap formation or it could also downregulate p-CDC42 at S71 and thereby promote the activity of CDC42 (the GTP-bound form) and facilitate cap formation

In sum, we showed for the first time that NLRP4E was predominant among NLRP4 family members within oocytes and that it was particularly involved in actin cap formation and spindle translocation toward the cap during meiosis I. The primary mechanism appears to be that p-NLRP4E activates SRC through phosphorylation at Tyr418, while p-SRC subsequently activates CDC42 (the GTP-bound form) by downregulating its phosphorylation at Ser71 (Fig. 8). In addition, p-SRC promotes actin cap formation. Further investigation is required to delineate further mechanistic details concerning the function of NLRP4E during oocyte meiosis.

### Supplementary Information

**Additional file 1: Table S1.** Commercial antibody information. **Table S2.** DNA templates for Nlrp4e UTR siRNA. Materials and methods.

**Additional file 2: Movie S1.** *NLRP4E-*KD oocyte was unable to progress into MII due to failure in homologous chromosome alignment and segregation. Related to Fig. 1K. Time-lapse movie of meiosis in WT or NLRP4E-KD oocytes shows that after 10 h of IVM, the chromosomes in WT oocyte have separated in a timely manner, and the first polar body extruded; in contrast, the chromosomes in NLRP4E*-*KD oocyte cannot align well at the metaphase plate and go through anaphase I into MII. The movie was taken 6 h after IVM (which corresponded to early metaphase) at a time interval of 30 min.

## Data Availability

The data that support the findings of this study are available from the corresponding author upon reasonable request.

## Abbreviations

AI: Anaphase I
Co-IP: Coimmunoprecipitation
GCs: Granulosa cells
GV: Germinal vesicle
GVBD: Germinal vesicle breakdown
HRP: Horseradish peroxidase
IF: Immunofluorescence
IP: Immunoprecipitated
IVF: In vitro fertilization
IVM: In vitro maturation
MALDI-TOF-MS: Matrix-assisted laser desorption/ionization time of flight mass spectrometry
MI: Metaphase I
MII: Metaphase II
Pb1: First polar body
siRNA: Small interfering RNA
TI: Meiosis telaphase I
NLRP: NACHT, LRR, and PYD domains-containing protein
SCMC: Subcortical maternal complex
GRIN1: G protein-regulated inducer of neurite outgrowth 1
GANO1: Guanine nucleotide binding protein, alpha O
RSK: Ribosomal protein S6 kinase alpha-1
RSHL1: Radial spoke head protein 6 homolog A
CPL: Cytoplasmic lattices
dTGN: Dispersed trans-Golgi network
IFN: Interferon
MATER: Maternal antigen that embryos require
NCLX: Mitochondrial Na^+^/Ca^2^^+^ exchanger
PND: Post-natal day
