# Supplementary material for: NLRP4E regulates actin cap formation through SRC and CDC42 during oocyte meiosis
Source: Cell Mol Biol Lett. 2024 May 10;29:68. doi: 10.1186/s11658-024-00580-y (PMC11088158; doi:10.1186/s11658-024-00580-y)
Supplement: Supplementary file 1 — Additional file 1: Table S1. Commercial antibody information. Table S2. DNA templates for Nlrp4e UTR siRNA. Materials and methods. [file 11658_2024_580_MOESM1_ESM.docx]

**NLRP4E Regulates Actin Cap Formation through SRC and CDC42 during Oocyte Meiosis**

**Additional materials and methods**

**Immunofluorescence staining**

Three steps were followed:

1). Oocytes permeation & fixation & blocking

After a brief wash in PBS/0.05% PVP (polyethylene-pyronidone, to prevent oocyte sticking), the oocytes were permeated in 0.5% Triton X-100/ PHEM for 5 min and quickly washed in PBS/PVP 3 times. Next, the oocytes were fixed in 3.7% paraformaldehyde/PHEM solution for 20 min, and then washed in PBS/PVP 3 times (10 min per wash). Finally, the oocytes were incubated in blocking buffer (1% BSA/PHEM, 100 mM glycine) at RT (room temperature) for 1 h.

2). Primary & secondary antibody incubation

Oocytes treated above were firstly incubated in blocking buffer containing primary antibody overnight (ON) at 4 °C, then washed in PBST (PBS with 0.05% Tween-20) 3 times (10 min per wash). Next, the oocytes were incubated in blocking buffer containing fluorescently-labeled secondary antibody (dilution ratio, 1:750) for 0.75 h at RT, then washed in PBST 3 times.

3). DNA staining and slide making

Oocytes treated above were stained in PBST 10 min with Hoechst 33342 (10 mg/ml, Sigma), washed in PBST, moved onto a sticky slide and cover by a drop (about 5 μl) of anti-fade mounting solution (0.5% propyl gallate, 0.1 M Tris-HCl, pH 7.4, 88% glycerol), then covered by coverslide (130 to 170 μm thick). To keep the oocyte structure intact, two pieces of double-stick tape (90 μm thick) were put between the coverslip and slide. Then the sample was ready for image taking on Confocal microscope (Andor, USA).

**Expression and Purification of recombinant NLRP4E and SRC proteins**

We cloned and expressed recombinant NLRP4E and SRC proteins using the Bac-to-bac system (Thermo Fisher). Simply, the SRC-EGFP-StrepII or NLRP4E-TagRFP-Flag sequence was cloned into pFastBacHTA (Thermo Fisher) and then transformed into DH10Bac Escherichia coli (E.coli, Vazyme) cells. The plasmid was isolated from DH10Bac cells by QI plasmid purification kit (QIAGEN) and transfected into Sf9 cells (Genetimes ExCell) with an optimized transfection reagent (Thermo) at 27 °C, where the first round of virus titer (baculovirus) was collected. Then Fresh Sf9 cells were infected with the first titer for 72 h, and the operation was repeated 1 to 2 times to increase the baculovirus titer. Then, 20 μl of the final virus titer was added to 250 ml of SFM900-II medium (Thermo) containing 5% FBS, and the proteins were expressed at 200 rpm, 27 °C on an orbital shaker.

Sf9 cells above containing the expressed proteins were then dispersed in lysis buffer (pH 7.0, with 50 mM Tris, 50µM ATP, 10% sucrose, 1 mM phenyl methyl sulfonyl fluoride (PMSF), 1 × protease inhibitor and phosphatase inhibitor) and fully cracked with a high-pressure cell cracking apparatus (Union Biotech). The cracked cells were centrifuged and the supernatant was incubated with 1 ml optimized Ni-NTA resin (QIAGEN) 1 h at 4 °C. The protein-bound resin is then loaded into a 5 ml column and washed with washing buffer (about four column volume, resuspension buffer with 40 mmol imidazole, no PMSF), and elution with an elution solution (resuspension buffer with 500 mmol imidazole, no PMSF). The eluted proteins were concentrated in a size-exclusion centrifuge column and the original buffer was replaced by BRB80 (pH 6.8 with KOH, 1 mM MgCl2, 80 mM HEPES, and 1 mM EGTA, 5 mM DTT, 50 μM ATP, and 10% glycerol). The proteins are equally divided and stored at -80 °C.

**Additional tables**

**Table S1. Commercial antibody information**

| **1^0^ or 2^0^** | **Antigen** | **Host** | **Clonality** | **Tag** | **Company info** | **Cat #** |
| --- | --- | --- | --- | --- | --- | --- |
| 1^0^ | GAPDH | Mouse | Mono- | None | YEASEN, China | 30201ES60 |
| 1^0^ | β-Actin | Mouse | Mono | None | Sigma, USA | A5316-100 |
| 1^0^ | β-Tubulin | Mouse | Mono | None | Santa Cruz, USA | sc-5274 |
| 1^0^ | Tubulin α | Mouse | Mono | None | Bioworld, USA | BS1699M |
| 1^0^ | Flag | Mouse | Mono | None | BBI Life science, China | D190828 |
|  | Strep II | Mouse | Mono | None | Yifeixue, China | YFMA0054 |
| 1^0^ | SRC | Rabbit | Poly | None | Proteintech, USA | 11097-1-AP |
| 1^0^ | p-SRC (Tyr418) | Rabbit | Poly | None | ProteinTech, USA | GTX24816 |
| 1^0^ | ARP3/ARP3B | Rabbit | Poly | None | Rosemont, IL, USA | 13822-1-AP |
| 1^0^ | GNAO1 | Rabbit | Poly | None | Rosemont, IL, USA | 12635-1-AP |
| 1^0^ | Cdc42 | Rabbit | Poly | None | Proteintech, USA | 10155-1-Ap |
| 1^0^ | p-Cdc42 (Ser71) | Rabbit | Poly | None | Bioss, China | bs-3369R |
| 2^0^ | Mouse IgG | Goat | Mono | HRP | Jackson ImmunoResearch, USA | 205-032-176 |
| 2^0^ | Rabbit IgG | Goat | Mono | HRP | Beyotime, China | A0208 |
| 2^0^ | Mouse IgG | Donkey | Mono | Cy2 | Jackson ImmunoResearch, USA | 715-225-150 |
| 2^0^ | Rabbit IgG | Donkey | Mono | Cy2 | Jackson ImmunoResearch, USA | 711-225-152 |
| 2^0^ | Rabbit IgG | Donkey | Mono | TRITC | Jackson ImmunoResearch, USA | 711-025-152 |
| 2^0^ | Human IgG | Donkey | Mono | Alexa 647 | Jackson ImmunoResearch, USA | 709-605-149 |

1^0^ or 2^0^, primary or secondary; Host, animals or cells where antibodies are produced; Clonality, monoclonal (mono-) or polyclonal (poly-); Tag, fluorescence tag or HRP tag; Company info., company information; Cat #, Catalog No.

**Table S2. DNA templates for Nlrp4e UTR siRNA**

| **Target Site** | **DNA templates** |
| --- | --- |
| *Nlrp4e* mRNA 62-82^1^ | Oligo1: GGATCCTAATACGACTCACTATAGAAGACGCATCTCTCTTCCTT^2^ |
|  | Oligo2: AAAAGGAAGAGAGATGCGTCTTCTATAGTGAGTCGTATTAGGATCC^2^ |
|  | Oligo3: GGATCCTAATACGACTCACTATAAAGGAAGAGAGATGCGTCTTC^2^ |
|  | Oligo4: AAGAAGACGCATCTCTCTTCCTTTATAGTGAGTCGTATTAGGATCC^2^ |
| *Nlrp4e* mRNA 3043–3063^1^ | Oligo1: GGATCCTAATACGACTCACTATAGAAGCAGAAACAAGGGTGGAT^2^ |
|  | Oligo2: AAATCCACCCTTGTTTCTGCTTCTATAGTGAGTCGTATTAGGATCC^2^ |
|  | Oligo3: GGATCCTAATACGACTCACTATAATCCACCCTTGTTTCTGCTTC^2^ |
|  | Oligo4: AAGAAGCAGAAACAAGGGTGGATTATAGTGAGTCGTATTAGGATCC^2^ |
| *Nlrp4e* mRNA 3191–3211^1^ | Oligo1: GGATCCTAATACGACTCACTATAGAGATACATACAGTTTCTTTA^2^ |
|  | Oligo2: AATAAAGAAACTGTATGTATCTCTATAGTGAGTCGTATTAGGATCC^2^ |
|  | Oligo3: GGATCCTAATACGACTCACTATATAAAGAAACTGTATGTATCTC^2^ |
|  | Oligo4: AAGAGATACATACAGTTTCTTTATATAGTGAGTCGTATTAGGATCC^2^ |
| *Nlrp4e* mRNA 3314–3334^1^ | Oligo1: GGATCCTAATACGACTCACTATAGAATGTTTATAATGACAGTTT^2^ |
|  | Oligo2: AAAAACTGTCATTATAAACATTCTATAGTGAGTCGTATTAGGATCC^2^ |
|  | Oligo3: GGATCCTAATACGACTCACTATAAAACTGTCATTATAAACATTC^2^ |
|  | Oligo4: AAGAATGTTTATAATGACAGTTTTATAGTGAGTCGTATTAGGATCC^2^ |
| Control^3^ | Oligo1: GGATCCTAATACGACTCACTATAGACCTACGCCACCAATTTCGT^2^ |
|  | Oligo2: AAACGAAATTGGTGGCGTAGGTCTATAGTGAGTCGTATTAGGATCC^2^ |
|  | Oligo3: GGATCCTAATACGACTCACTATAACGAAATTGGTGGCGTAGGTC^2^ |
|  | Oligo4: AAGACCTACGCCACCAATTTCGTTATAGTGAGTCGTATTAGGATCC^2^ |

^1^ The numbers are the starting and ending position of the target sites in mouse *Nlrp4e* mRNA (NM_001004194.2 in NCBI).

^2^ two pairs of DNA oligos are needed for for each double-stand siRNA. Oligo 2 is complementary with oligo 1 except an "AA" overhang at 5'; Oligo 3 is complementary with oligo 4 except an "AA" overhang at 5'. In each oligo, gene-specific sequences are underlined, other sequences are for recognition and binding by T7 RNA polymerase.

^3^ Control siRNA does not target to any mRNA sequence in mouse.
